# Synthesis and Characterization of Novel Anion Exchange Membranes Based on Semi-Interpenetrating Networks of Functionalized Polysulfone: Effect of Ionic Crosslinking

**DOI:** 10.3390/polym13060958

**Published:** 2021-03-20

**Authors:** Sydonne Swaby, Nieves Ureña, María Teresa Pérez-Prior, Alejandro Várez, Belén Levenfeld

**Affiliations:** Department of Materials Science and Engineering and Chemical Engineering, IAAB, Universidad Carlos III de Madrid, Avda. Universidad, 30, Leganés, E-28911 Madrid, Spain; sswaby@ing.uc3m.es (S.S.); murena@ing.uc3m.es (N.U.); maperezp@ing.uc3m.es (M.T.P.-P.); alvar@ing.uc3m.es (A.V.)

**Keywords:** polysulfone, semi-interpenetrating network, ionic crosslinking, anion exchange membrane, AEMFC

## Abstract

In this work, anion exchange membranes based on polymer semi-interpenetrating networks were synthesized and characterized for the first time. The networks are composed of sulfonated polysulfone and 1-methylimidazolium-functionalized polysulfone crosslinked covalently with *N*,*N*,*N*′,*N*′-tetramethylethylenediamine (degree of crosslinking of 5%). In these membranes, sulfonic groups interact electrostatically with cationic groups to form an ionic crosslinking structure with improved alkaline stability. The effect of the ionic crosslinking on the thermal, chemical, mechanical, and electrochemical behavior of membranes was studied. These crosslinked membranes containing sulfonated polysulfone showed higher thermal stability, with a delay of around 20 °C in the onset decomposition temperature value of the functional groups than the crosslinked membranes containing free polysulfone. The tensile strength values were maintained above 44 MPa in all membranes with a degree of chloromethylation (DC) below 100%. The maximum ionic conductivity value is reached with the membrane with the highest degree of chloromethylation. The chemical stability in alkaline medium of the conducting membranes also improved. Thus, the ionic conductivity variation of the membranes after 96 h in a 1 M potassium hydroxide (KOH) solution is less pronounced when polysulfone is replaced by sulfonated polysulfone. So, the ionic crosslinking which joins both components of the blends together, improves the material’s properties making progress in the development of new solid electrolyte for polymeric fuel cells.

## 1. Introduction

In recent decades there has been a growing demand for alternative energy as a result of different factors like climate change. This fact implies the need to reduce the emission of greenhouse gases and the limited availability of fossil fuels, whose price and accessibility are strongly influenced by social, political, economic, and geographical factors [1]. In order to reduce these factors, fuel cells are a very promising option, particularly due to their high efficiency and low emissions [2,3,4,5]. Therefore, many works are being carried out to optimize this technology through both experimental results and modelling [6,7]. Electric vehicles and portable devices are some of their applications [4,8,9].

Fuel cells based on polymer electrolytes such as proton- and anion- exchange membrane fuel cells (PEMFCs and AEMFCs, respectively) operate at a temperature lower than 100 °C, and their use is therefore favored from environmental and economical points of view [10,11]. Recently, the research and development of AEMFCs are increasing over PEMFCs because of some advantages. Firstly, it is possible to use catalysts that do not contain platinum such as Ni-Cr and Ag [11,12,13]. Also, there is a wide variety of materials available for the manufacture of AEMFCs. Thus, in addition to hydrogen, different fuels, such as hydrazine or ammonia can be used [11,14]. However, anion exchange membranes (AEMs) have low alkaline stability at high temperatures, and low OH^−^ conductivity in comparison to the PEMs. This last is due mainly to the fact that the diffusion coefficient of the protons is four times higher than that of the hydroxide groups [10,15,16]. Therefore, to overcome some of these limitations which affect negatively the performance of the fuel cell, the chemical structure of the AEM must be optimized in order to obtain improved properties.

An AEM is constituted by a polymeric backbone which sustains the dimensional stability of the membrane, and a cationic functional group which is responsible of the ionic conductivity [1,10,11,14,17]. Generally, functionalized polymers exhibit lower dimensional stability than non-functionalized ones [9,14]. In this context, to provide dimensional stability and improve the mechanical and chemical properties of the functionalized polymers used, crosslinking of polymer chains can be considered a simple option. The interaction linkage of the polymer chains would result in a three-dimensional structure with higher rigidity with respect to the free polymer. If the polymeric chains have good dimensional stability, higher degrees of functionalization can be achieved, and the resulting membrane will show greater ionic conductivity.

Different crosslinking agents are used in the synthesis of polymers for AEMs. For example, 1,4-diazabicyclo-[2,2,2]-octane (DABCO) has a high volumetric structure that provides higher resistance to the polymer [2]. Pérez-Prior et al. [18] prepared a series of membranes based on polysulfone (PSU) with DABCO, which acted as a functional group and as a crosslinking agent. Crosslinked membranes showed greater capacity to control the water absorption than that observed for non-crosslinked ones, and higher alkaline stability associated with the spatial structure of DABCO which protects membrane from Hoffmann elimination. *N*,*N*,*N*′,*N*′-tetramethylethylenediamine (TMEDA) is widely used as a crosslinking agent. The presence of two nitrogen atoms in their structure could improve the ionic conductivity of the AEM and could stabilize positive loads and prevent polymer degradation [19].

Currently, the synthesis of interpenetrating polymer networks (IPNs) composed of two crosslinked polymers, and semi-interpenetrating polymer networks (sIPNs) in which only one polymer is crosslinked are being developed to prepare membranes for diverse applications like nanofiltration [20], reverse osmosis [21], and also in fuel cells as solid electrolytes [22]. In this line, AEMs for fuel cells applications were also prepared from IPNs and sIPNs [14]. Thus, Qiao et al. [23] synthesized AEMs which exhibited long-term stability in hot water (60 °C) and hot alkali solution (6 M KOH at 80 °C). Such stability is attributed to the formation of interpenetrated networks between chemical crosslinks of both components of the blend, i.e., poly (vinyl alcohol) (PVA) and poly (acrylamide-co-diallyldimethylammonium chloride (PAADDA). These systems combine the properties of the blends as well as the benefits of crosslinking [24]. He et al. [25] developed sIPNs based on copolymers of Polysulfone Udel^®^ (PSU) functionalized with benzyltrimethylammonium (BTMA) and styrene-divinylbenzene for AEMs. The percentage of water absorption capacity was reduced and the OH^−^ conductivity at room temperature increased.

Xue et al. [17] prepared a series of sIPN composed of copolymers of quaternized poly (styrene) (PS) and crosslinked poly (2,6-dimethyl-1,4-phenyleneoxide) (PPO), obtaining structures which can effectively protect the cations from being attacked by hydroxide ions. Thus, these AEMs presented higher chemical stability under fuel cell operation conditions. Another example of this type of blends were reported by Pan et al. [26]. They prepared mechanically tough and chemically stable AEMs from rigid-flexible semi-interpenetrating networks composed of quaternized PPO and a crosslinked poly (ethylene glycol) (PEG). The resulting membranes were composed by a rigid and ion-conductive component, and a second component which is hydrophilic, crosslinked, and flexible, favoring the flexibility of the material and thus improving its mechanical behavior.

In all of these systems, the two components of the blends interact through Van der Waals forces which are weak intermolecular forces. So, the use of ionic polymers that can interact electrostatically is proposed to strengthen these interactions favoring thus the dimensional stability of the membrane [27,28]. Li et al. [3] prepared AEMs from blends of ionic polymers to promote ionic crosslinking between backbones. They used a basic imidazolium-functionalized poly (ketone ether ether ketone) (PEEK) as a conducting component and sulfonated poly (ketone ether ether ketone) (SPEEK) in acid form which allows the formation of ionic crosslinking. An improvement on the mechanical properties of the resulting membranes was observed, increasing 30% of the Young’s Modulus and tensile strength (TS) by the increasing the content of the SPEEK of the blend and the DS of SPEEK. In a similar work, Hande et al. [29] studied the effect that the acid-base interaction has on the properties of SPEEK mixed with an unconjugated diamine (3,3′-dichloro-4,4′-diaminodiphenylmethane). The thermomechanical properties of the resulting material, as well as the dimensional and oxidative stability, were improved. Xu et al. [28] explained with the formation of the ionic crosslinking between poly (aryl ether ketone) (PAEK) functionalized with imidazoles (1,2-dimethylimidazole and 1-butyl-2-methylimidazole) and SPEEK, the increase in TS and the efficient swelling control of the membranes synthesized.

In this study, AEMs based on sIPNs were synthesized for the first time. 1-Methylimidazolium-functionalized PSU (MIm-PSU) covalently crosslinked with TMEDA sulfonated PSU compose the blend. To ensure the compatibility of the two components of the blend, PSU was used as a polymer backbone in both cases. The ionic conductivity of the membrane was attributed to MIm-PSU covalently crosslinked with the diamine. The presence of crosslinked polymers in the blend was expected to enhance the dimensional stability of the membrane. On the other hand, sulfonated polysulfone (SPSU) was used as a macromolecular crosslinker through their sulfonic groups, which electrostatically interact with the cationic groups of the conducting polymer to form an ionic crosslinking structure. The effect that ionic crosslinking exerts on thermal, chemical, mechanical, and electrochemical properties of the prepared membranes was studied.

## 2. Materials and Methods

### 2.1. Materials and Reagents

Polysulfone Udel^®^ (PSU, 22,000 g·mol^−1^), 1,2-dichloroethane (DCE, 99.8%), trimethylsilyl chlorosulfonate (TMSCS, 99.0%), chloroform (99.0%), tin (IV) chloride (99.0%), 1-methylimidazole (≥99.0%), *N*,*N*,*N*′,*N*′-tetramethylethylenediamine, paraformaldehyde (95.0–100.5%), *N,N*-Dimethylformamide (DMF-d_7_, (≥99.5%) and dimethyl sulfoxide-d_6_ (DMSO-d_6_, 99.9%) were supplied by Sigma-Aldrich (Munich, Germany). Chlorotrimethylsilane (≥99.0%) and 1-methyl-2-pyrrolidone (NMP, 99.0%) were purchased from Merck (Darmstadt, Germany) and Alfa Aesar (Kandel, Germany) respectively.

### 2.2. Synthesis of Anion Exchange Membranes

#### 2.2.1. Synthesis of Chloromethylated Polysulfones

**Figure 1:**
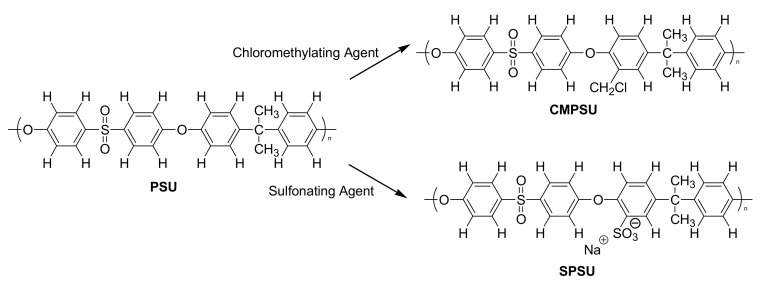
Synthesis of chloromethylated polysulfone (CMPSU) and sulfonated polysulfone (SPSU).

Chloromethylated polysulfone (CMPSU) was prepared by chloromethylation reaction of PSU with a paraformaldehyde/chlorotrimethylsilane mixture as chloromethylating agent and tin (IV) chloride (SnCl_4_) as catalyst [18,30] (Figure 1). In a 500 mL flask, 5.00 g of PSU was dissolved in 250 mL of chloroform. Once the PSU was dissolved, paraformaldehyde (6.79 g) was added, followed by SnCl_4_ (0.27 mL), and finally chlorotrimethylsilane (28.64 mL) was incorporated by using a dropwise funnel. Once all of the reagents had been added, the flask was covered to keep it at 55 °C with constant stirring. The reaction time varied from 48 to 72 h as a function of the degree of chloromethylation (DC) desired. The resulting CMPSU was precipitated in methanol. The chloromethylated polymer was kept in a dry environment.

#### 2.2.2. Synthesis of Sulfonated Polysulfones

Sulfonated polysulfone (SPSU) was synthesized according to published data [31] and the scheme in Figure 1. In a three-neck round bottom flask (250 mL), 5.00 g of PSU was dissolved in 30 mL of DCE at room temperature under Argon atmosphere. After dissolving the polymer, TMSCS used as a sulfonating agent, dissolved in DCE, was added. SPSU was prepared by using 1:1 PSU:TMSCS molar ratio. The reaction was maintained for 24 h. SPSU obtained was precipitated in a 0.1 M solution of sodium hydroxide (NaOH) and the polymer was kept in a dry environment. Figure 1 shows a schematic route to obtain both CMPSU and SPSU.

#### 2.2.3. Functionalization of PSU with 1-Methylimidazole

**Figure 2:**
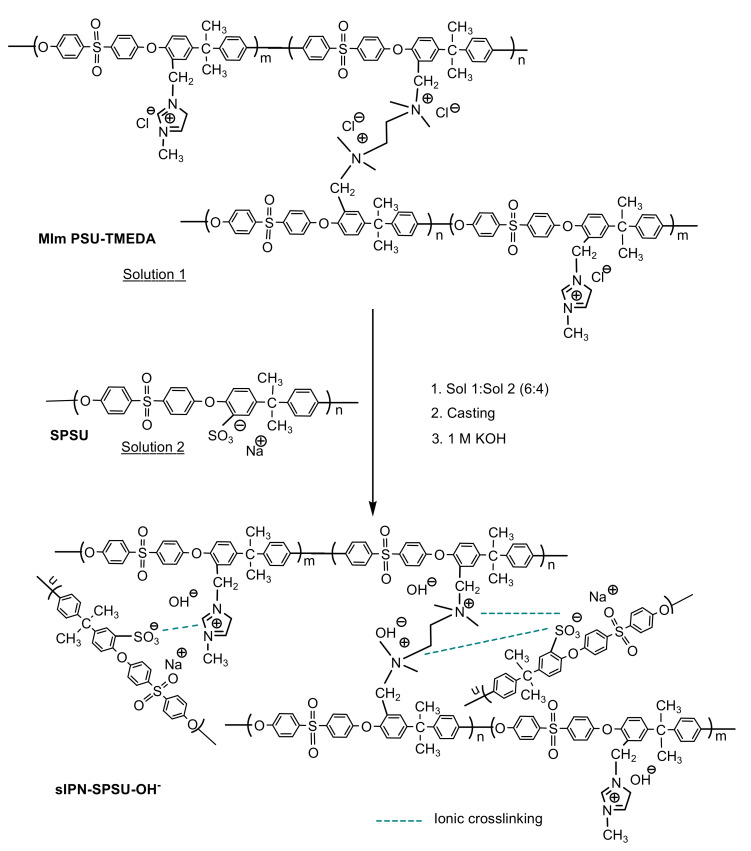
Synthesis of semi-interpenetrating polymer network (sIPN)-SPSU-OH^−^ membranes.

1-Methylimidazolium-functionalized polysulfone (MIm-PSU) was obtained by means of nucleophilic substitution reaction between chloromethylated polysulfone and 1-methylimidazole (MIm) (Figure 2). The CMPSU was dissolved in 6 mL of NMP in a round bottom flask and then 1-methylimidazole was added. The volume of this reagent varied depending on the percentage of functionalization we wanted to obtain; totally functionalized with 1-methylimidazole, 100%, or partially functionalized, 95%, where some of the chloromethyl groups remain free to be able to react with the crosslinking agent. The reaction was maintained for 3 h at 60 °C and constant stirring.

#### 2.2.4. Preparation of Polymer Blends: Ionic Crosslinking

Once the MIm-PSU was obtained, the TMEDA, acting as crosslinking agent was added to the flask and kept under stirring for 15 min at room temperature. The amount of TMEDA was added to obtain a theoretical crosslinked percentage of 5% (Solution 1). Also, non-crosslinked polymer was synthesized in other to study the effect of covalent crosslinking in the membranes. After this time, the free polymer (PSU or SPSU) previously dissolved in NMP (Solution 2) was added to the flask. It was kept for 5 min under stirring at room temperature. Solutions 1 and 2 were mixed in a 6:4 molar ratio for a total polymer mass of 0.9 g. This polymer ratios were chosen according to a previous work where the experimental conditions were optimized [32].

#### 2.2.5. Preparation of Membranes

Membranes were prepared from these three-dimensional networks by means of casting in NMP (Figure 2). The content of the flask (solution 1 + solution 2) was poured into a Petri dish. To evaporate de solvent, the temperature of the oven was increased gradually until 80 °C four 24 h under vacuum [28]. Membrane in the Cl^−^ form was immersed in a 1 M KOH solution for 48 h to replace Cl^−^ by OH^−^. Finally, the membrane containing OH^−^ as counter anions was repeatedly rinsed with deionized water until the pH of the residual water was neutral. The thickness of the resulting membranes was around 100 μm. In this work, we prepared membranes with the DC of 75% and 143% and DCl of 0% and 5%. The synthesized membranes will be denoted as sIPN_x,y_-PSU and sIPN_x,y_-SPSU where x and y correspond to the degree of chloromethylation (DC) and degree of covalent crosslinking (DCl), respectively. The non-crosslinked membrane was used as a reference. 

### 2.3. Measurements

#### 2.3.1. ^1^H-NMR

The structural analysis of the non-crosslinked membranes as well as the intermediate species involved in the synthesis was carried out by proton nuclear magnetic resonance spectroscopy (^1^H-NMR). The measurements were performed on a Bruker Avance (Billerica, MA, USA) DPX-300 ^1^H-NMR spectrophotometer (300 MHz). The solvent used was DMSO-d_6_, DMF-d_7_ and tetramethylsilane (TMS) was used as a reference.

#### 2.3.2. Thermogravimetric Analysis (TGA)

The thermal stability of the membrane samples (30.0 mg) was studied in a Perkin-Elmer Pyris STA 6000 equipment (Waltham, MA, USA) and in a Pyris TGA1 instrument from Perkin-Elmer (Waltham, MA, USA) in the temperature range 30–850 °C under nitrogen and air atmospheres at a heating rate of 10 °C·min^−1^. The thermal characterization of the membranes was carried out taking into account the onset decomposition temperature (*T_OD_*) and the fastest decomposition temperature (*T_FD_*). Both parameters are defined as the temperature at which the weight loss begins and the temperature of the maximum weight loss rate, respectively. The value of *T_FD_* was obtained from the derivative thermogravimetric curve.

#### 2.3.3. Mechanical Properties

The mechanical properties were evaluated with a dynamo mechanical analyzer TA Instruments (DMA Q800) (New Castle, DE, USA) by performing Stress-Strain tests in uniaxial tension mode at a constant temperature of 30 °C. These tests were conducted in controlled force mode with a force ramp of 0.3 N·min^−1^ up to 18 N and a frequency of 1 Hz. The initial static force was imposed with a value of 0.15 N. The dimensions of the specimens were 8 × 2 mm^2^ and around 133 µm of thickness. The membranes in Cl^−^ form were dried at 60 °C in an oven for 24 h. For each membrane, three samples were tested.

#### 2.3.4. Water Uptake (WU%)

The water uptake of the membrane sample was evaluated by measuring the weight difference between the fully hydrated membrane and completely dry membrane at room temperature. To calculate the WU%, the membrane was taken in dry chlorinated form and immersed in a 1 M KOH solution for 48 h to replace the Cl^−^ with OH^−^, and thus, obtain the membrane in its hydrated form. The chlorinated and hydrated samples were weighted in order to determine the WU% as shown below with Equation (1):

(1) $$WU\%=\frac{m_{hyd(OH)}-m_{dry(Cl)}}{m_{dry(Cl)}}\times 100$$

where *m_dry_*_(*Cl*)_ is the mass of the dried membrane in the Cl^−^ form, and *m_hyd_*_(*OH*)_ the mass of the wet membrane in the OH^−^ form.

#### 2.3.5. Ion-Exchange Capacity (IEC)

The IEC of the membranes were determined by using a standard acid-base titration. The membrane in the chloride form was immersed in a 1 M KOH solution for 24 h to exchange the ion to obtain the membrane in the OH^−^ form. Next, the membrane in the hydroxide form was immersed in 20 mL of a 0.1 M HCl solution for 48h. A volume (15 mL) of the resulting acid solution was titrated by using a 0.1 M KOH solution. After the titration, the membrane was dried in the oven to determine the dry weight of the Cl^−^ form. The IEC is calculated as follows:

(2) $$IEC=\frac{n_{i(H^{+})}-n_{f(H^{+})}}{m_{dry(Cl)}}$$

where *n_i_*_(*H*_*^+^*_)_ and *n_f_*_(*H*_*^+^*_)_ are the initial and final moles of H^+^ in the HCl solution, respectively.

#### 2.3.6. Ionic Conductivity

The ionic conductivity of the membranes was measured by electrochemical impedance spectroscopy (EIS). A Solartron 1260 equipment (Farnborough, UK) was used as an impedance analyzer with a Solartron 1287 electrochemical interface in a frequency range between 10^−1^ and 10^6^ Hz. The conductivity cell used consists of two half-cells containing the liquid electrolyte (KOH) connected to each other through a 1.1 cm radius hole where the membrane was placed [33]. The configuration consists of four electrodes, of which two graphite electrodes act as working electrodes and two saturated Ag/AgCl electrodes act as reference electrodes. Electrochemical measurements were made at different concentrations of the liquid electrolyte (between 10^−4^ and 10^−1^ M). Prior to ionic conductivity measurements, the chlorinated membranes were immersed in a 1 M KOH solution for 24 h, and consequently these were measured in OH^−^ form. The ionic conductivity of the membrane was determined using the following equation:

(3) $$\sigma_{m}=\frac{L}{R_{m}\times A}$$

where *σ_m_* (S cm^−1^) is the ionic conductivity, *L* (cm) the thickness, *A* (cm^2^) the experimental area, and *R_m_* (Ω) is the measured resistance value of the membranes. It was obtained from the impedance plot which was analyzed with the Z-View impedance analyzer software.

The influence of the temperature in the ionic conductivity of the membranes was studied by using a KMF 115 climate chamber (Binder GmbH). Conductivity measurements were made in a temperature range between 30 and 80 °C, using an electrolyte concentration of 10^−3^ M. The activation energy (*E_a_* in kJ·mol^−1^) was calculated using Arrhenius equation in its linearized form:

(4) $$ln\sigma =\ln\sigma_{0}-\frac{E_{a}}{R\cdot T}$$

where *σ*, *σ_o_*, *R*, and *T* are the conductivity of hydroxide in S·cm^−1^, the pre-exponential factor, the constant of the ideal gases in J·(mol·K)^−1^ and the temperature in K, respectively.

#### 2.3.7. Alkaline Stability

The alkaline stability of membranes in aqueous medium was tested through the variation of their ionic conductivity throughout the time. The membranes were immersed in a 1 M KOH solution at room temperature for 48, 72 and 96 h. After this time, the ionic conductivity at room temperature of the samples was determined. The liquid electrolyte concentration was 10^−3^ M.

## 3. Results and Discussion

### 3.1. Strategy

Semi-interpenetrating polymer networks were characterized by the penetration on a molecular scale of at least one of the networks by at least some of the linear or branched polymers [34]. These materials combine the properties of both constituents, although the average is not simple. In a preliminary work, we prepared AEMs from semi-interpenetrating polymer networks based on methylimidazolium-functionalized PSU crosslinked in turn with TMEDA, which was responsible for the OH^−^ conduction and PSU as a linear polymer [32]. We observed an improvement in thermal, mechanical, and alkaline stability of the membranes. This article extends our previous work by proposing polymer networks where PSU is replaced by sulfonated PSU, where the formation of stronger intermolecular forces ion-ion between sulfonic and imidazolium groups would be favored, thus improving the union between the two components of the blend.

These ionic polymers, SPSU and MIm-PSU, could interact efficiently through ion-ion interactions which imply an energy of around 250 kJ·mol^−1^. This value was higher than that observed in the networks comprising only one ionic polymer. Interactions between the two components of the system were weak, mainly dipole-dipole forces or, in some cases, hydrogen bonds. In all cases the energy involved waws lower than 20 kJ·mol^−1^ which indicate a poor union of the mixture [35].

On this matter, the present paper reports a systematic study of the effect of ionic crosslinking on the properties of sIPNs based on polysulfone.

### 3.2. Structural Characterization

#### 3.2.1. Synthesis of Methylimidazolium-Functionalized Polysulfone Crosslinked with TMEDA

The functionalization reaction of polysulfone with 1-methylimidazole followed by covalent the crosslinking with TMEDA takes place in three steps: (i) chloromethylation of PSU; (ii) functionalization reaction with 1-methylimidazole; and (iii) crosslinking of polymer chains with TMEDA.

**Figure A1:**
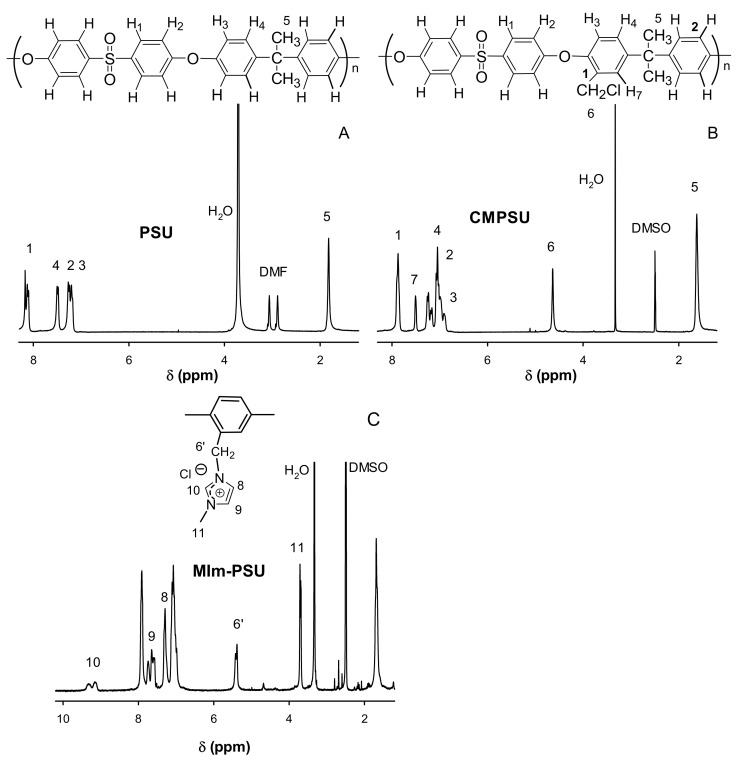
^1^H-NMR Spectra of PSU (**A**), CMPSU (**B**), and MIm-PSU (**C**) membranes (DMF-d_7_ and DMSO-d_6_).

The chloromethylation reaction of PSU is an electrophilic substitution reaction. It preferably occurs at carbon in the α position with respect to the carbon next to the oxygen atom of the PSU repeating unit. This carbon has the highest electronic density, and because of this, the substitution reaction at this position seems to be favorable. When all the carbons in α position are substituted, the chloromethyl groups also are anchored in the β carbons obtaining a disubstituted polymer. The ^1^H-NMR spectrum of CMPSU (Figure A1) reveals that the chloromethylation reaction of the PSU successfully occurred [3]. We can confirm this fact because of: (i) the appearance of the characteristic peak of the protons of the CH_2_Cl groups at a displacement *δ* = 4.6 ppm; and (ii) the displacement of the peak associated with the proton of the carbon located in α position with respect to the carbon containing the chloromethyl group [18,30,36]. The degree of chloromethylation of PSU is calculated with the peak associated with the protons of the chloromethyl groups, and the peak of the aromatic protons (see Appendix A), which is not shifted when the reaction takes place, according to published work [32]

The functionalization with 1-methylimidazole is carried out from CMPSU by a nucleophilic substitution reaction with the chloromethyl groups of the polymer. Non-crosslinked polymer is analyzed by ^1^H-NMR. The ^1^H-NMR spectrum of 1-methylimidazolium-functionalized polysulfone (Figure A1) verifies that the substitution of the chloromethyl groups has been occurred. Thus, the peak corresponding to the protons of the methylene group in the CMPSU spectrum is displaced in the spectrum of the polymer functionalized with MIm in accordance with a previous work [32].

Covalent crosslinking of the polymer chains is carried out with TMEDA through the chloromethyl groups that remain free after the functionalization reaction with 1-methylimidazole.

#### 3.2.2. Sulfonation of PSU

**Figure 3:**
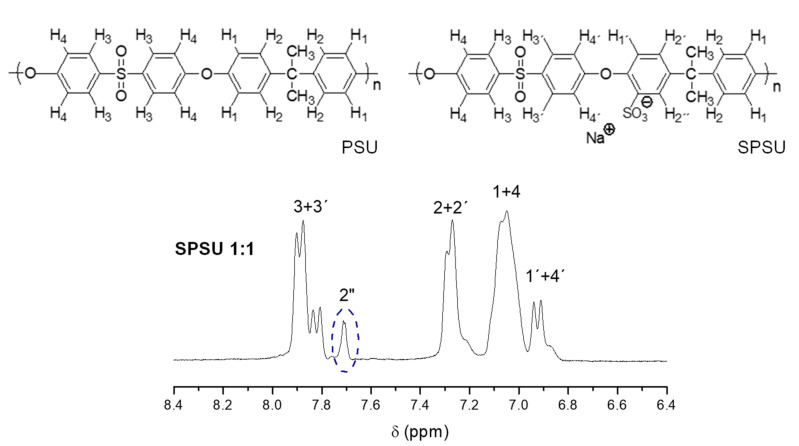
Proton nuclear magnetic resonance spectroscopy (^1^H-NMR) spectrum of SPSU 1:1 (DMSO-d_6_).

The sulfonation reaction was successfully confirmed using ^1^H-NMR spectroscopy. Figure 3 shows the spectra of PSU and SPSU in the range of chemical shift corresponding to aromatic protons (6.4 ppm < δ < 8.4 ppm) whose signals vary after the sulfonation reaction of PSU occurs.

As can be seen in Figure 3, the peaks corresponding to the protons H_1_ and H_4_ in the spectrum of SPSU appear at 7.0 ppm, whereas protons H_2_ and H_3_ are related to the peaks at 7.3 and 7.9 ppm. The area of the peak at a shift of 7.7 ppm, denoted as 2″, corresponds to the benzene carbon ring located in *α* position with respect to the carbon that contains the sulfonic group. So, the sulfonation reaction of PSU successfully occurred in accordance with published data [31,37].

The degree of sulfonation (DS) of PSU was determined by ^1^H-NMR, as described by Iojoiu et al. [31], by using the Kopf equation. Thus, the value of DS for the SPSU used in the present work (polymer:sulfonating agent ratio of 1:1) was 44%. Lower DS values make the formation of the ionic crosslinked structure difficult, whereas the presence of a large number of negative charges due to sulfonic groups would neutralize the positive charges of imidazolium groups decreasing the OH^−^ conductivity of membranes [28].

### 3.3. Thermogravimetric Analysis (TGA)

**Figure 4:**
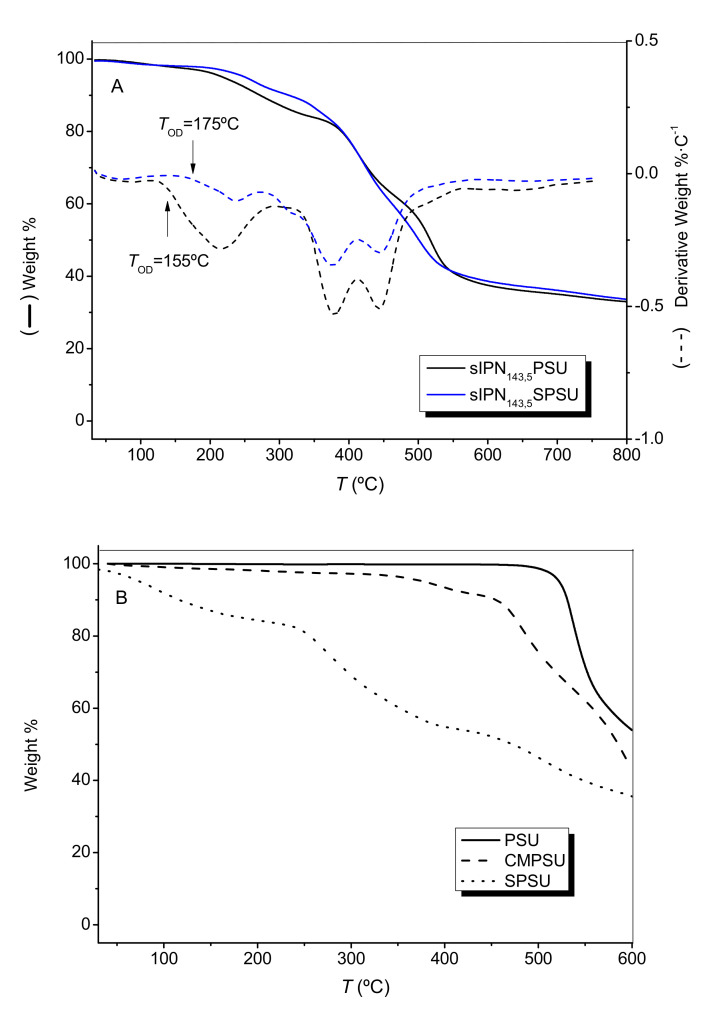
(**A**) Thermogravimetric curves of sIPN_143,5_-PSU and sIPN_143,5_-SPSU under nitrogen atmosphere. Weight % and derivative weight of the membranes. (**B**) Thermogravimetric analysis (TGA) curves of PSU, CMPSU and SPSU under air atmosphere.

The thermal stability of the membranes has been studied by thermogravimetric analysis in the temperature range from 30 to 850 °C. As an example, Figure 4A shows the thermograms (weight and derivative weight versus temperature) of sIPN_143,5_-PSU and sIPN_143,5_-SPSU. TGA curves of the polymers isolated along the synthesis are also shown in Figure 4B.

In both curves, three mass losses were observed. The first weight loss between 40 and 100 °C is associated with the retained water in the membranes. The second loss between 160 and 250 °C corresponds to the decomposition of the functional groups such as MIm [38], sulfonic groups in the case of the SPSU, and TMEDA used as a crosslinking agent. Around 400–450 °C, there was another weight loss associated with the degradation of the polymeric backbone [18,30]. Membrane sIPN_143,5_-PSU exhibited values of *T_OD_* and *T_FD_* of 155 °C and 257 °C, respectively. Li et al. [3] observed a similar behavior for an AEM functionalized with 1-methylimidazole where described the decomposition of imidazolium groups about 190 °C. The membrane sIPN_143,5_-SPSU showed a value of *T_OD_* of 175 °C and *T_FD_* of 257 °C. Membranes composed of SPSU present sulfonic groups (negative ions) interacting with both the imidazolium groups and the TMEDA diamine (positive ions) by forming an ionic crosslinking structure which prevent these ionic groups from thermal decomposition. As a consequence of this, the thermal stability of the membranes at a temperature of around 200 °C (with a delay of 20 °C in the *T_OD_*), which is associated with the degradation of these groups, was enhanced [3,29]. 

### 3.4. Mechanical Properties

**Figure 5:**
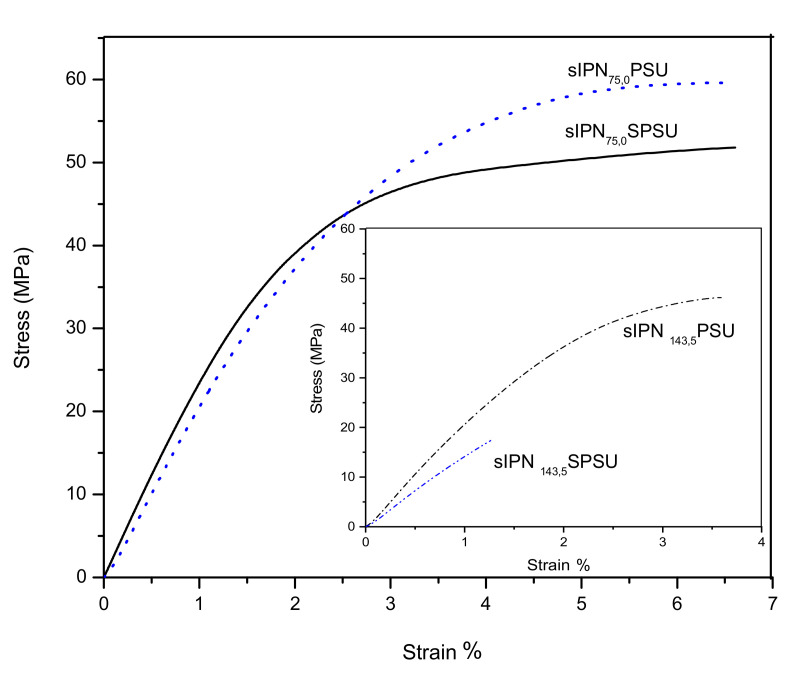
Stress-Strain curves of sIPN_75,0_-PSU (-----) and sIPN_75,0_-SPSU (·····) in the external chart and sIPN_75,5_-SPSU (-·-·-·-) and sIPN_143,5_-SPSU (-··-··-) in the internal chart.

To evaluate the mechanical properties of these membranes, a dynamo-mechanical analysis was performed. As an example, Figure 5 shows the stress-strain curves of some of the most representative membranes dry in the Cl^−^ form. Thus, the effect that degree of chloromethylation, covalent crosslinking, and ionic crosslinking has on the mechanical behavior of membranes was evaluated through the pairs sIPN_75,0_-PSU and sIPN_75,0_-SPSU (Figure 5) and sIPN_75,5_-SPSU and sIPN_143,5_-SPSU (Figure 5 inset). Table 1 shows the tensile strength and elongation at break (ε) values obtained from the stress-strain curves of the membranes.

From these results it can be extracted that membranes with different degree of chloromethylation (75% and 143%) exhibit values for both parameters TS and *ε* clearly different. Thus, the TS values of the membranes sIPN_75,5_-SPSU and sIPN_143,5_-SPSU are 44 ± 2 and 12 ± 3 MPa, respectively. Highly functionalized membranes exhibit low tensile strength values. This fact could be associated with the decrease of molecular weight of the polymer chains due to the chloromethylation reaction [30]. In the case of the membranes with DC = 75%, the TS values were maintained above 44 MPa because of: (i) the low number of functional groups in the polymer (below 100%), this fact implies that 75 out of 100 repeating units contain one functional group, and then the mechanical stability of the resulting membrane is more similar to the starting polymer than that measured for highly functionalized polysulfones; and (ii) the presence of free PSU in the blend enhances the mechanical properties by comparing with functionalized PSU with imidazolium groups.

On the other hand, the covalent crosslinking of polymer chains by TMEDA decreases the TS and *ε* values of the resulting membranes, although the effect that covalent crosslinking has on the membrane mechanical properties is less significant than the DC. For instance, the obtained values for sIPN_75,0_-PSU and sIPN_75,5_-PSU were 58 ± 2 and 49 ± 3 MPa, respectively. This trend was also observed when membranes based on crosslinked poly (vinylbenzyl chloride) for AEM were synthetized by Lu et al. [39]. The TS values of these membranes decrease from 19.4 to 14 MPa (conventional uniaxial tensile test at a strain rate of 0.5 mm min^−1^). The *ε* values of non-crosslinked membranes show higher standard deviation than those obtained for crosslinked ones. This fact could be associated with the presence of two free polymers (MIm-PSU and SPSU) in the blend without covalent crosslinking which act independently.

Regarding the interaction between the sulfonic groups of SPSU and the cationic ones such as imidazolium and ammonium functionalities, the TS obtained does not vary significantly when SPSU is incorporated into the network. This effect is observed for membranes with high and low DC values. An increase in the tensile strength of the membranes with SPSU associated with the electrostatic interactions between ionic groups could be expected. However, the sulfonation reaction is aggressive and breaks the polymer chains [37], thus decreasing the molecular weight of SPSU with respect to that of PSU. So, the SPSU acting as free polymer in the blend does not impart the expect rigidity to the network. The TS of the PSU membrane is around 60 MPa [32] whereas the value for SPSU is clearly lower, of around 10 MPa [37].

In view of these results, the TS values obtained for the sIPN network shown in this work are higher than those observed for other non-crosslinked AEMs. Thus, Yang et al. studied the mechanical properties of functionalized PSU with trimethyl ammonium (TMA) in the OH^−^ form (DC of 85%), reaching a TS value of around 10 MPa [40]. In another study, Gong et al. [41] reported values of 18 MPa of TS and 7% *ε* at covalent crosslinked PSU membranes that were used as component of a IPN system.

Table 1 displays the *ε* percentages of the membranes. The values obtained in all the membranes here studied do not exceed a value around 10%, thus revealing the low flexibility of these membranes. This behavior could be associated with the nature of both polymer backbone and functional group, which is characterized by their limited flexibility. Values previously reported vary between 5% and 18% for similar membranes [42]. As has been studied, the mechanical properties of the polymers are affected after any chemical modification such as chloromethylation and sulfonation reactions. In this case, both components of the blend were functionalized and its was clearly observed that the membranes maintained their tensile strength with a small difference in the elongation at break associated with the homogeneous casting of this novel complex sIPN which are promising to be applied in AEMs.

### 3.5. Water Uptake

The water absorption capacity of membranes has direct effects on the dimensional stability, and the conductivity of the membranes [43,44]. High values of this parameter can cause an important loss of the dimensional stability, making the material weak and unstable. Table 2 shows the WU% values for a series of membranes with different DC and DCl values.

As expected, the most influential parameter in the WU is the degree of chloromethylation. Highly functionalized membranes exhibit greater values of WU%. The WU% of the membranes studied with DC of 75% are around 10%, while for membranes with DC of 143% these values are doubled (in the range 22–25%). So, the capability of the membranes to absorb water clearly increases with the percentage of functional groups linked to the polymer backbone. The ionic nature of these groups increases the polarity of the polymer favoring thus their solvation with water molecules. This behavior so pronounced has been previously observed in AEMs functionalized with amines like TMA [45], DABCO [18], and imidazoliums and their derivatives [46,47]. Likewise, the presence of SPSU in the network does not cause the WU% values of the membranes to vary significantly; this result can be associated with: (i) the low DS of the PSU used in the network; and (ii) the small ratio of SPSU in the blend. So, the degree of chloromethylation is the variable that controls the capacity of membranes to absorb water. In addition, all values obtained are low, and this fact favors the dimensional stability of the membranes.

### 3.6. Ion Exchange Capacity

This parameter, together with water uptake, closely affects the ionic conductivity of membranes. The IEC values increase with increasing DC, since there are more exchangeable groups anchored to the polymer. Thus, IEC varied from 0.94 to 1.16 mmol g^−1^when the DC increased from 75% to 143%. In general, IEC values achieve an increase in presence of covalent crosslinking established through TMEDA. This is due to cations formed with covalent crosslinking favoring the ion exchange capacity of the material, and when SPSU is added to the blend, the IEC decreases slightly. As a similar result obtained by Xu et al. [28], the ionic crosslinking limits the IEC of the membranes because an electrostatic interaction between the two components of the blends. In this study, the ionic crosslinking produces a similar effect in the membranes obtained in this work.

### 3.7. Ionic Conductivity

**Figure 6:**
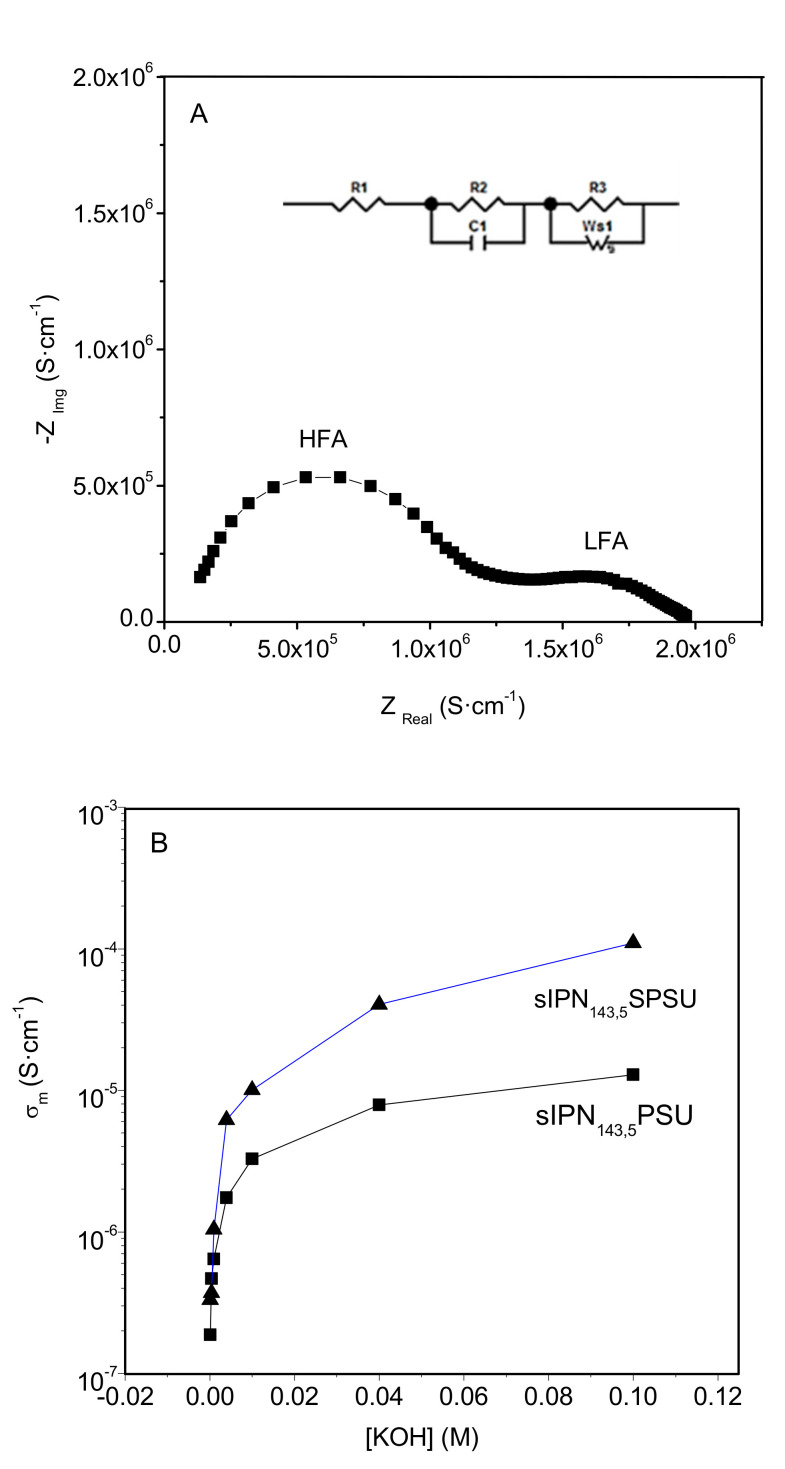
(**A**) Impedance plot of sIPN_75,5_-PSU-OH membrane and (**B**) Evolution of the OH^−^ conductivity of sIPN_143,5_-PSU-OH and sIPN_143,5_-SPSU-OH membranes as a function of the KOH concentration. Measurements were performed at room temperature.

The ionic conductivity of the membranes was determined by electrochemical impedance spectroscopy. Figure 6A shows, as an example, the Nyquist diagram (−Z_img_ vs. Z_real_) obtained for the sIPN_75,5_-PSU membrane in the hydroxide form. As expected, two semicircles were observed. The first semicircle at high frequency (HFA) is related to the contribution of the liquid electrolyte (diluted KOH solution) in contact with the membrane, while the low frequency arc (LFA), which is distorted, is associated with the membrane containing the liquid solution [30,37]. The high frequency semicircle does not intercept the origin of the graph, this indicates the presence of a resistive element in series with the two other processes [33]. Similar Nyquist plots were obtained in the rest of the membranes studied in the present work. Table 2 shows the ionic conductivity values of the membranes synthesized. Measurements were carried out at room temperature and [KOH] = 0.1 M.

The evolution of membrane ionic conductivity (*σ_m_*) with KOH concentration for sIPN_143,5_ -PSU-OH and sIPN_143,5_-SPSU-OH membranes is shown in Figure 6B. The conductivity of these two membranes increased with KOH concentration over the entire concentration range used, from 10^−4^ M to 10^−1^ M, being more pronounced for the membrane containing sulfonated polysulfone. This behavior was observed in all membranes studied here [18,30].

As shown in Table 2, the ionic conductivity increased one order of magnitude when the DC value varied from 75% to 143%. Thus, the highest *σ_m_* value was achieved for the membranes with DC of 143%. The conductivity is, then, strongly influenced by the degree of functionalization of polysulfone, as was observed in previous works [18,30]. However, the effect of the covalent crosslinking on the ionic conductivity was not clearly observed in these membranes. This fact could be due to the low percentage of covalent crosslinking linked to the high complexity of our network, and regarding to the effect that ionic crosslinking has on *σ_m_* values, we observed two different behaviors. For higher degrees of chloromethylation (DC = 143%) membranes with SPSU have higher ionic conductivity than those with PSU, whereas membranes with a low percentage of functional groups exhibit better properties when PSU acts as a free polymer in the blend.

**Figure A2:**
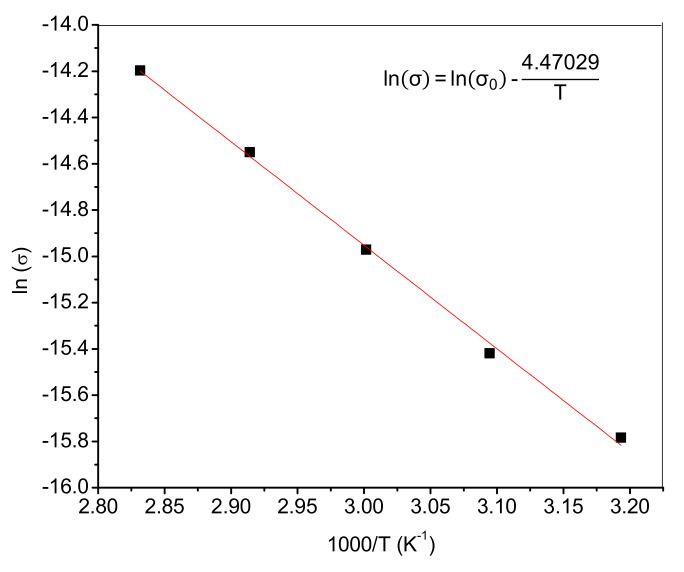
Arrhenius plot for the sIPN_143,5_-SPSU membrane.

The influence of temperature on *σ_m_* was also studied. The experimental data obey Arrhenius law and the apparent activation energy (*E_a_*) associated with the OH^−^ transport through the membrane was easily obtained from the slope of the ln *σ_m_* *versus* 1/*T* plot (Figure A2). The OH^−^ conduction process through membranes based on sIPNs containing PSU and SPSU had an activation energy of 37 and 20 kJ·mol^−1^, respectively. So, membranes containing an ionic crosslinking structure have higher difficulty to transport hydroxide ions. The ionic interaction between the two components of the blend seems to distort the morphology of the channels through which the hydroxide ions circulate, making them narrower and thus impeding their conduction [3]. In addition, these values are higher than those reported in the literature for anion-exchange membranes (11 kJ·mol^−1^) [25] which reveals the impediment that the polymer network has on the membrane ionic conduction.

### 3.8. Alkaline Stability: Effect of Ionic Crosslinking

The stability of the membranes in alkaline medium compromises the lifetime of the membranes and, therefore, the fuel cell performances. This is due to the cationic group being susceptible to be degraded in basic medium and, consequently, the ionic conductivity of the membrane decreasing [2]. The alkaline stability of the membranes has been determined through the variation of the ionic conductivity that occurs when the membranes were treated in alkaline solutions for a time ranging from 24 to 96 and 168 h defined as Δ*σ*,

(5) $$\Delta \sigma =\frac{\sigma_{f}-\sigma_{i}}{\sigma_{i}}\times 100$$

where *σ_f_* and *σ_i_* are the final and initial conductivity, respectively.

**Figure 7:**
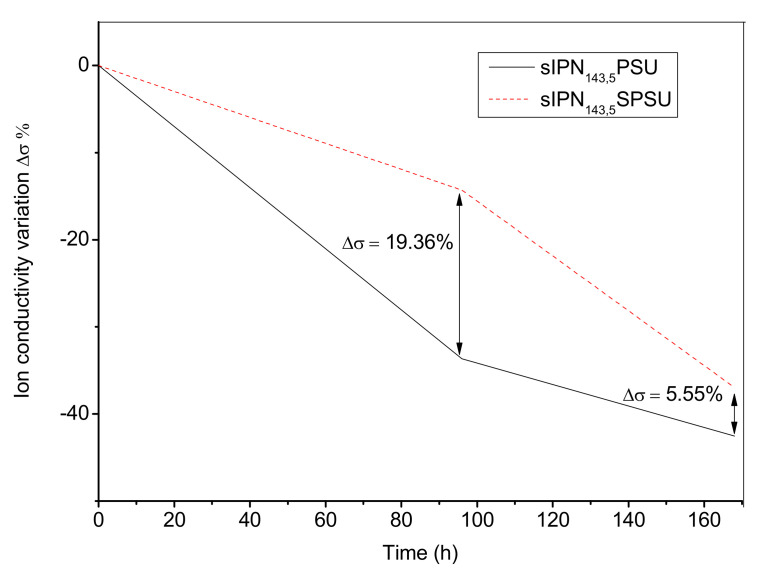
Ionic conductivity variation of the membranes sIPN_143,5_-PSU and sIPN_143,5_-SPSU after 96 and 168 h in a 1 M KOH solution at room temperature.

In all of the membranes studied, independently of the functionalization degree, a decrease in ionic conductivity was observed when they were treated in 1 M KOH solution after 96 and 168 h at room temperature. As an example, in Figure 7 the variation of the ionic conductivity observed for two membranes (sIPN_143,5_-PSU and sIPN_143,5_-SPSU) is shown.

The effect of the ionic crosslinking in membranes with SPSU was evaluated (Table 3). It can be observed that the loss of ionic conductivity was less pronounced on membranes containing SPSU for all DC values. The ionic crosslinking in the sIPNs preserved the membrane conductivity [25]. As is shown in Table 3, for higher DC (143%) the ionic conductivity loss was observed after 96 h. However, for lower DC values (75%) the decrease of conductivity started after 168 h.

A similar behavior was observed in a previous work for similar polymers [28]. Adding SPSU to the sIPNs improve the alkaline stability of the membranes. Therefore, the membranes obtained in this work are a good candidate for AEMs.

## 4. Conclusions

A novel series of AEMs were synthesized from mixtures composed of PSU functionalized with 1-methylimidazolium and crosslinked with TMEDA and sulfonated polysulfone. An ionic crosslinking structure was established between the two components of the blend. The effect of ionic crosslinking on the membrane’s behavior was studied. The thermal stability slightly increased with respect to the membrane without ionic crosslinking (about 20 °C delayed). The outstanding tensile strength observed in the membranes with PSU stayed in the same range of values reached with the membrane with SPSU for chloromethylation degrees below 100%. Although the sulfonation reaction caused the polymer chains to break and worsened the mechanical stability, the ionic crosslinking compensated for this loss of stability. Thus, the tensile strength values were maintained above 44 MPa in all membranes with a DC of 75%. The IEC of these membranes was favored with the degree of chloromethylation and the percentage of covalent crosslinking, however it slightly decreased with the ionic crosslinking, as previously reported in the bibliography. Ionic conductivity of membranes was strongly influenced by the degree of chloromethylation of the polymer. The highest value was achieved in the highest functionalized membranes (DC = 143%). The activation energy of the conduction process with membranes presenting ionic crosslinking is 37 kJ·mol^−1^, this value is higher than that obtained with membranes containing PSU as a free polymer (20 kJ·mol^−1^). Therefore, the ion–ion interactions established between the ionic polymers form a network in which the membrane conduction process is not favored. However, it is noteworthy that the chemical stability of the membranes in a basic medium increased when PSU was replaced by SPSU. Results reveal that the degradation of the functional groups is less favored when these cationic groups interact by means of strong ion–ion molecular forces with the sulfonic groups attached to PSU in the three-dimensional network. In brief, the ionic crosslinking established between the components of the mixture improves the thermal and the alkaline stability of the ion exchange membranes synthesized in this work. Therefore, it would be a good proposal for improvement in obtaining these materials for application as solid electrolytes in fuel cells.

Future work should consider the preparation of sIPNs composed of two different polymer backbones, to aim at a clear nanophase separation which improves the electrochemical properties of the resulting membranes. Moreover, the synthesis of functionalized polymers with more stable nitrogen-based functional groups in alkaline media would reinforce the electrostatic interactions between these cationic groups and sulfonic ones, thus favoring the ionic crosslinking structure.

## Figures and Tables

**Table 1:** Tensile strength and elongation at break (ε) determined from the stress-strain curves of the membranes in the Cl^−^ form. Standard deviation is also indicated.

Membrane	TS (MPa)	*ε* %
sIPN_143,5_-PSU	15 ± 4	1.8 ± 0.9
sIPN_143,5_-SPSU	12 ± 3	2.6 ± 0.4
sIPN_75,5_-PSU	49 ± 3	5.3 ± 0.6
sIPN_75,5_-SPSU	44 ± 2	3.5 ± 0.4
sIPN_75,0_-PSU	58 ± 2	7 ± 3
sIPN_75,0_-SPSU	52 ± 2	10 ± 2

**Table 2:** Values of water uptake (WU%) and ionic conductivity (*σ_m_*) of the membranes at room temperature.

Membrane	WU%	*σ_m_* (mS·cm^−1^) ^a^
sIPN_143,5_-PSU	22.0	1.29 × 10^−2^
sIPN_143,5_-SPSU	25.0	1.10 × 10^−1^
sIPN_143,0_ SPSU	-	-
sIPN_75,5_-PSU	8.8	1.24 × 10^−3^
sIPN_75,5_-SPSU	5.0	2.21 × 10^−4^
sIPN_75,0_-PSU	8.4	7.32 10^−2^
sIPN_75,0_-SPSU	11.0	1.15 × 10^−4^

^a^ [KOH] = 0.1 M.

**Table 3:** Ionic conductivity variation of the membranes after 96 and 168 h in a 1 M KOH solution.

Membrane	Δ*σ* % (96 h)	Δ*σ* % (168 h)
sIPN_143,5_-PSU	−33.67	−42.52
sIPN_143,5_-SPSU	−14.31	−36.97
sIPN_75,5_-PSU	0	−11.97
sIPN_75,5_-SPSU	0	−11.88

## Abbreviations

PEMFC	Proton exchange membrane fuel cell
AEMFC	Anion exchange membrane fuel cell
AEM	Anion exchange membrane
PEM	Proton exchange membrane
DABCO	1,4-diazabicyclo-[2,2,2]-octane
PSU	Polysulfone
TMEDA	*N*,*N*,*N*′,*N*′-tetramethylethylenediamine
IPN	Interpenetrating polymer network
PVA	Poly (vinyl alcohol)
PAADDA	Poly (acrylamide-co-diallyldimethylammonium chloride
sIPN	Semi-interpenetrating polymer network
BTMA	Benzyltrimethylammonium
PS	Poly (styrene)
PPO	Poly (2,6-dimethyl-1,4-phenyleneoxide)
PEG	Poly (ethylene glycol)
SPEEK	Sulfonated poly (ketone ether ether ketone)
TS	Tensile strength
PEEK	Poly (ketone ether ether ketone)
PAEK	Poly (aryl ether ketone)
MIm-PSU	1-Methylimidazolium-functionalized polysulfone
DCE	1,2-dichloroethane
TMSCS	Trimethylsilyl chlorosulfonate
DMF-d_7_	*N*,*N*-Dimethylformamide-d_7_
DMSO-d_6_	Dimethyl sulfoxide-d_6_
NMP	1-methyl-2-pyrrolidone
CMPSU	Chloromethylated polysulfone
DC	Degree of chloromethylation
SPSU	Sulfonated polysulfone
MIm	Methylimidazole
DCl	Degree of covalent crosslinking
^1^H-NMR	Proton nuclear magnetic resonance spectroscopy
TMS	Tetramethylsilane
TGA	Thermogravimetric analysis
*T_OD_*	Onset decomposition temperature
*T_FD_*	Fastest decomposition temperature
WU%	Water uptake
IEC	Ion-exchange capacity
EIS	Electrochemical impedance spectroscopy
DS	Degree of sulfonation
TMA	Trimethyl ammonium
HFA	High frequency arc
LFA	Low frequency arc

## Appendix A

The ^1^H-NMR spectra of PSU, CMPSU and MIm-PSU membranes are shown in Figure A1. PSU shows the peaks associated with phenyl and methyl groups at δ = 6.9–7.9 ppm and δ = 1.7 ppm, respectively [30]. From the ^1^H-NMR spectrum of CMPSU, the DC value is easily calculated by using this equation: DC = [2A(H_6_)/A(H_1_)] × 100 where A(H_6_) and A(H_1_) are the integral area of the H_6_ and H_1_ peaks, respectively. The conversion from the chloromethyl group into a methylimidazolium group is also observed in the ^1^H-NMR spectrum of MIm-PSU. The presence of MIm groups in the sIPN membrane is confirmed by the appearance of a new characteristic peak at 3.8 ppm (H_7_) corresponding to the proton of methyl group. The formation of imidazolium increases the acidity of the proton located between the two nitrogen atoms leading to the downshift of its signal until 9.2 ppm (H_8_). The complete conversion from a chloromethyl group to an imidazolium one is confirmed by the shift of the proton signal of the methylene from 4.6 ppm (H_6_) in CMPSU to 5.5 ppm (H_6′_) in the MIm-PSU membrane.

## Appendix B

The effect that temperature has on the ionic conductivity of membranes is also studied. Measurements were performed in an ionic conductivity cell containing a 10^−3^ M KOH solution. Temperature was controlled in a climate chamber. Figure A2 shows the Arrhenius plot for the sIPN_143,5_-SPSU membrane.
